# Identification of Nrf2-responsive microRNA networks as putative mediators of myocardial reductive stress

**DOI:** 10.1038/s41598-021-90583-y

**Published:** 2021-06-07

**Authors:** Justin M. Quiles, Mark E. Pepin, Sini Sunny, Sandeep B. Shelar, Anil K. Challa, Brian Dalley, John R. Hoidal, Steven M. Pogwizd, Adam R. Wende, Namakkal S. Rajasekaran

**Affiliations:** 1 Molecular and Cellular Pathology, University of Alabama at Birmingham, Birmingham, AL USA; 2 Department of Biomedical Engineering, University of Alabama at Birmingham, Birmingham, AL USA; 3 Huntsman Cancer Center-Genomic Core Facility, University of Utah, Salt Lake City, UT USA; 4 Division of Cardiovascular Medicine, Department of Medicine, University of Utah, Salt Lake City, UT USA; 5 Division of Pulmonary Medicine, Department of Medicine, University of Utah, Salt Lake City, UT USA; 6 Comprehensive Cardiovascular Center, Department of Medicine, University of Alabama at Birmingham, Birmingham, AL USA; 7 Division of Molecular and Cellular Pathology, Department of Pathology, Center for Free Radical Biology, The University of Alabama at Birmingham, BMR2 Room 533, 901 19th Street South, Birmingham, AL 35294-2180 USA

**Keywords:** Cardiology, Cardiovascular biology, Gene ontology, Gene regulatory networks

## Abstract

Although recent advances in the treatment of acute coronary heart disease have reduced mortality rates, few therapeutic strategies exist to mitigate the progressive loss of cardiac function that manifests as heart failure. Nuclear factor, erythroid 2 like 2 (*Nfe2l2*, *Nrf2*) is a transcriptional regulator that is known to confer transient myocardial cytoprotection following acute ischemic insult; however, its sustained activation paradoxically causes a reductive environment characterized by excessive antioxidant activity. We previously identified a subset of 16 microRNAs (miRNA) significantly diminished in *Nrf2*-ablated (*Nrf2*^−/−^) mouse hearts, leading to the hypothesis that increasing levels of *Nrf2* activation augments miRNA induction and post-transcriptional dysregulation. Here, we report the identification of distinct miRNA signatures (i.e. “reductomiRs”) associated with *Nrf2* overexpression in a cardiac-specific and constitutively active *Nrf2* transgenic (ca*Nrf2*-Tg) mice expressing low (TgL) and high (TgH) levels. We also found several *Nrf2* dose-responsive miRNAs harboring proximal antioxidant response elements (AREs), implicating these “reductomiRs” as putative meditators of *Nrf2*-dependent post-transcriptional regulation. Analysis of mRNA-sequencing identified a complex network of miRNAs and effector mRNAs encoding known pathological hallmarks of cardiac stress-response. Altogether, these data support *Nrf2* as a putative regulator of cardiac miRNA expression and provide novel candidates for future mechanistic investigation to understand the relationship between myocardial reductive stress and cardiac pathophysiology.

## Introduction

During ischemia–reperfusion injury, generation of reactive oxygen and nitrogen species results in oxidative stress, which in turn, perturbs cardiac structure and function through calcium mishandling, inflammatory signaling and extracellular matrix degradation^1–3^. However, the findings from clinical studies have largely discredited the efficacy of antioxidant therapies^4,5^. Furthermore, our laboratory has identified the presence of a “reductive stress” wherein aberrant induction of antioxidant response element (ARE)-dependent antioxidant genes produces pathological cardiac hypertrophy and dysfunction^6–8^. While the deleterious consequences of reductive stress appear highly-conserved^9,10^, the transcriptional and post-transcriptional mechanisms of the myocardial redox milieu remain unknown.

Among the mechanisms known to regulate postnatal heart function, microRNAs (miRNAs) are a class of short (~ 22 nucleotide) RNAs that post-transcriptionally regulate mRNA stability and translational efficiency, often in a tissue-specific manner^11^. While miRNAs are necessary for physiologic cardiac function and development^12,13^, many have been found to be dysregulated in the failing heart^14–16^. Specifically, miRNAs have been directly linked to the structural and functional deficits in cardiac function^17^. Nevertheless, it remains unclear whether ARE-linked miRNAs contribute to cardiac pathogenesis.

As a transcriptional activator of cis-regulatory AREs^18^, nuclear factor erythroid 2-related factor 2 (*Nfe2l2*, a.k.a. *Nrf2*) plays a critical role in regulating cardiac redox status. Transient *Nrf2* signaling is cardioprotective immediately following ischemic insult^19^, but chronic transactivation of AREs causes reductive stress and cardiac dysfunction^20,21^. We have recently shown that *Nrf2* deficiency (*Nrf2*^−/−^) inhibits the expression of several miRNAs in the heart^22^, but the genome-wide impact of reductive stress on miRNA expression remains unknown.

In this investigation, we identify a miRNA signature for reductive stress to gain insight into potential biomarkers and/or effectors of this novel pathological phenomenon in the heart. The cardiomyocyte-specific and constitutively-active *Nrf2* transgenic mouse model (CaNrf2-Tg) was used to conduct a multi-omics analysis of *Nrf2*-dependent and ARE-bearing miRNAs, which we term “reductomiRs”. Our use of both mRNA-seq and small RNA sequencing (miRNA-seq) in ca*Nrf2* low (TgL) and high-expressing (TgH) mouse lines reveals a distinct signature of transgenic *Nrf2* dose-responsive miRNAs linked to a number of suppressed cardiac genes under pro-reductive and reductive stress conditions^23^. Collectively, this analysis uncovers several novel miRNA candidates for which future mechanistic studies will investigate the interplay between post-transcriptional regulatory responses and redox state in the myocardium of *Nrf2*-Tg mice.

## Methods

### Animals

Our method for establishing the cardiac-specific constitutively active *Nrf2* transgenic mouse model (ca*Nrf2*-Tg) has been described previously^23^. Briefly, cDNA encoding a truncated *Nrf2* protein lacking the Neh2 domain was ligated into an α myosin heavy chain (αMHC) expression vector, the plasmid backbone was digested, and the αMHC-ca*Nrf2* insert was used for pronuclear injection. Transgenic low (TgL) and transgenic high (TgH) founders were determined using ca*Nrf2* primer sets in real-time qPCR which compared relative transgene expression to endogenous *Nrf2* mRNA, and transgenic mice were back-crossed onto the C57BL/6J background for six generations. For expression analyses, sex-matched male and female TgL, TgH and non-transgenic (NTg) littermate heart tissue was harvested at 6 months of age. All the animal studies including non-invasive cardiac imaging were conducted in accordance with the Guide for Care and Use of Laboratory Animals developed by the National Research Council at the National Institutes of Health (NIH). The Institutional Animal Care and Use Committee (IACUC#14-10160) at the University of Alabama at Birmingham has approved the study, which was carried out in compliance with the Guidelines for Animal Research Reporting of In Vivo Experiments (ARRIVE).

### GSH-NEM immunofluorescence assay

To measure the redox status of Tg & NTg mouse hearts, we performed immunofluorescence using anti-GSH staining^24^. The mouse was anesthetized (2% isoflurane) and the heart was perfused in situ with 10 mM PBS-N-Ethylamaleimide (NEM) before sacrificing. The heart tissue sections (5 µM) were incubated in ethanol containing 10 mM NEM for 30 min and washed three times with PBS prior to blocking. Slides were incubated at 4 °C overnight with the primary antibody mouse anti-Glutathione:N-Ethylamaleimide adduct (1:500 v/v; EMD Millipore Corp., Billerica, MA, US). Following the incubation with the secondary Ab conjugated to Alexa-Flour 488 and washing, imaging was performed using a Fluorescence microscope (Olympus BX43). The intensity of green fluorescence representing glutathione (GSH) was calculated using Image-J (NIH) in 6 images from 3 hearts per group.

### Non-invasive cardiac imaging

Two-dimensional trans-thoracic echocardiography was performed in NTg and Tg mice at 6–7 months of age using the Vevo2100 system (Fujifilm Visual Sonics Inc., Ontario, Canada). In brief, animals were prepared for imaging as previously described^25^. Using a 38-MHz mechanical transducer, strain analysis in parasternal long-axis were acquired under isoflurane (1.5–2.0%) anesthesia. Structural and functional (systolic and diastolic) measurements were obtained using Vevo lab 3.1 software.

### RNA and miRNA sequencing analysis

Details of the R coding scripts and other bioinformatics tools used in the current study are available as online Supplemental Methods and GitHub data repository: https://github.com/mepepin/ReductomiRs. High-throughput RNA-and miRNA-sequencing and analysis were performed at the University of Utah. Alignment of reads to the mm10 genome was accomplished using NovoAlign (Singapore, Malaysia), raw counts generated using Samtools^26^, and differential gene expression performed using DESeq2^27^ (1.18.1) within the R (3.4.2) statistical computing environment as described previously^28^. Due to limited sample sizes (n = 3–4), dispersion estimates were first determined via maximum-likelihood, assuming that genes of similar average expression strength possess similar dispersion, as previously described^27^. Gene-wise dispersion estimates were then shrunken according to the empirical Bayes approach, providing normalized count data for genes proportional to both the dispersion and sample size. Differential expression was determined from normalized read counts via Log_2_ (fold-change) using the Wald test followed by Bonferroni-adjusted *P-*value (i.e. *Q-*value) for each aligned and annotated gene. Statistical significance was assessed with unpaired two-tailed Bonferroni-adjusted *P-*value (*Q-*value) < 0.05 used to identify differentially-expressed candidate genes, with a lower-stringency statistical threshold used for pathway enrichment (*P* < 0.01). Differentially expressed genes (DEGs) were generated with biological significance assumed when |Log_2_FoldChange|> 1 with normalized count sum > 1.

### RNA isolation and real-time quantitative polymerase chain reaction

Total RNA was extracted from 10 to 25 mg of the heart’s apex using QIAzol lysis reagent (Qiagen, Cat. 79306) and the miRNeasy mini kit (Qiagen, Cat. 217004). After confirming sample purity through the NanoDrop One Spectrophotometer (ThermoFisher Scientific), the miScript II RT Kit including HiSpec Buffer (Qiagen, Cat. 218161) was used to reverse transcribe 1–2 μg RNA. A 10 μl reaction containing 20 ng cDNA template and miScript primer assays (Qiagen, Cat. miRs-361-3p:MS00025718, 491-5p:MS00002541, 34b-3p:MS00011900, 1983:MS00016884, 671-5p:MS00064385, 671-3p:MS00012467 5121:MS00043071, 215-5p:MS00001918, 200c-3p:MS00001827, 155-5p:MS00001701) and SYBR Green® (Qiagen Cat. 218075) was amplified according to the manufacturer’s instructions in a Roche LightCycler 480 (Roche Life Science). Sequences used to quantify relative transgene abundance were: forward primer (mCa*Nrf2*-MHC-F), ACTTTACATGGAGTCCTGGTGGGA; reverse primer (mCa*Nrf2*-MHC-R), AGGCATCTTGTTTGGGAATGTGGG. Endogenous *Nfe2l2/Nrf2* transcript levels were measured using forward primer (m*Nrf2*-F), CTGAACTCCTGGACGGGACTA; reverse primer (m*Nrf2*-R), CGGTGGGTCTCCGTAAATGG. For each qPCR assay, equal amounts of cDNA were loaded for all control and experimental samples. Relative expression was determined according to the 2^−ΔΔCt^ method using small nucleolar RNAs *Snord42b* or *Snord72* as housekeeping genes.

### Data visualization

Functional and network gene set enrichment analysis (GSEA), along with curated literature-supported candidate upstream regulators, were performed using *Enrichr*, an interactive web-based tool for compiling multiple bioinformatics databases^29^. Within this software, Wikipathways^30^ and KEGG^31^ enrichment as well as ChIP Enrichment Analysis (ChEA) were done both on the individual datasets and as a combined comparative analyses to determine overlapping enriched pathways and associated transcriptional regulators, respectively. Heatmap generation and hierarchical clustering were performed using *pheatmap* package (1.0.8) within R, and VennPlex^32^ was used to create the Venn diagrams and determine overlapping gene lists.

### Statistics

All data are represented as mean ± standard error of the mean (SEM) unless otherwise indicated. Statistical significance was determined using unpaired Student’s *t-*tests or, where appropriate, one-way (ANOVA) with Tukey post-test for multiple comparisons. Statistical analyses and data visualization were completed using GraphPad Prism (GraphPad Software, San Diego, CA) and R software, version 3.4.2 (R Foundation for Statistical Computing, Vienna, Austria). Statistical significance was assigned at the *P* < 0.05 level for two-way comparisons.

### Consent for publication

All authors verified the content and approved the final version for submission and publication.

## Results

### Evidence of reductive stress and pathologic cardiac remodeling in Ca*Nrf2*-transgenic mice

**Figure 1:**
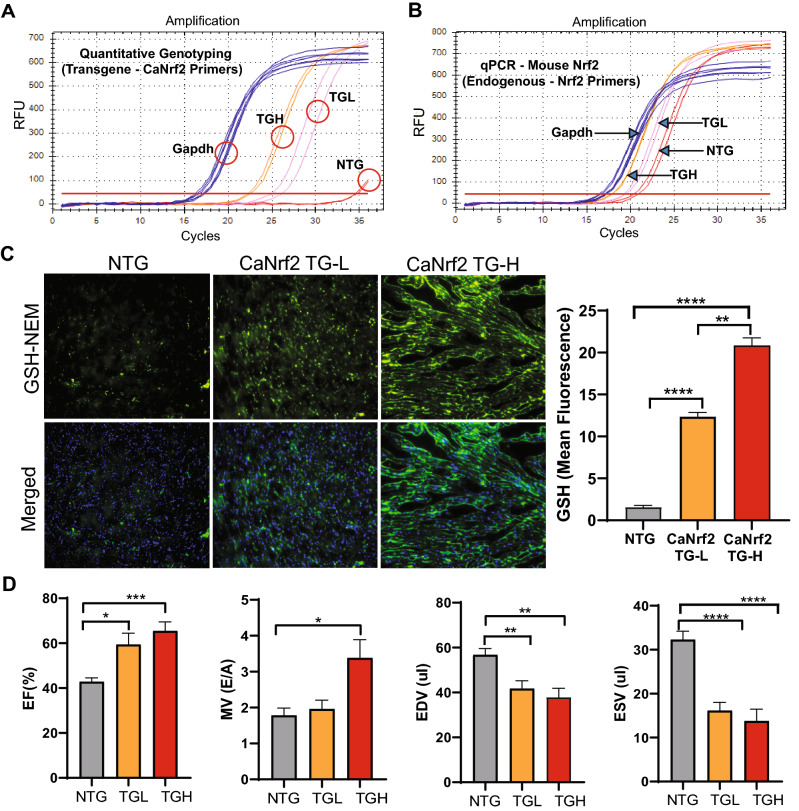
Differential expression of transgene, myocardial glutathione, structural and functional remodeling in caNrf2 mice. Real-time qPCR-based quantitation of transgene (CaNrf2) expression in transgenic low (TgL) high (TgH) and non-transgenic (NTg) hearts using (**A**) genotyping primers (recognizes caNrf2) and (**B**) endogenous Nrf2 primers vs Gapdh (n = 4/group). (**C**) Representative immunofluorescent (IF) images and quantification of **N-Ethylmaleimide-Glutathione adducts** (green) and DAPI (blue) in heart sections of NTg, TgL and TgH mice (n = 3/group). (**D**) Two-dimensional trans-thoracic echocardiography (para-sternal long axis strain analysis) was used to estimate ejection fraction (EF%), mitral valve (early, E to late, A ratio) filling, end diastolic volume (EDV) and systolic volume (ESV) (n = 10/group).

The concept myocardial reductive stress was first described in a mouse model of human cardiac disease^8^. However, the underlying molecular mechanisms remained unknown until the recent development of unique mouse models to study this phenomenon^25,33^. We recently described that constitutive activation of the transcription factor, *Nrf2*/NFE2L2, resulting in a dose-dependent increase of glutathione and other antioxidants, establishes a reductive and hyper-reductive redox condition in the heart^33^. Furthermore, a chronic hyper-reductive condition (i.e. “reductive stress”) was shown to cause pathological cardiac remodeling and diastolic dysfunction^25^. Here, we sought to identify a redox-responsive miRNA signature associated with reductive- and hyper-reductive-conditions in ca*Nrf2* transgenic low (TgL) and high (TgL) hearts. Quantitative genotyping in TgL and TgH mice indicated dose-dependent transgene expression (respective Ct values) when compared to NTg, which did not amplify a PCR product for the primers that recognize only the truncated transgene (*caNrf2*) (Fig. 1A). Interestingly, qPCR using a primer that recognizes endogenous mouse *Nrf2*, but not *caNrf2*, also showed a dose-wise expression pattern consistent with reports of *Nrf2* transcriptional auto-up-regulation (Fig. 1B)^34^. As *Nrf2* is well-characterized to promote glutathione biosynthesis^35^, we performed immunofluorescent staining of glutathione-N-ethylmaleimide (GSH-NEM) adducts in TgL and TgH to confirm the biochemical effects of dose-dependent transgene expression (Fig. 1C). These results confirm our previous studies employing kinetic-based spectrophotometric measurements for glutathione in Ca*Nrf2*-Tg hearts^33^. To determine the consequences of *Nrf2* expression on cardiac function, echocardiographic assessments (strain analysis) were performed (n = 10/group), which provided clear evidence of diastolic dysfunction in TgL and TgH relative to NTg mice (Fig. 1D). Taken together, these results suggest that *Nrf2* expression leads to both reductive conditions and pathological cardiac remodeling.

### Dose dependent effects of *Nrf2* on cardiac miRNA expression

**Figure 2:**
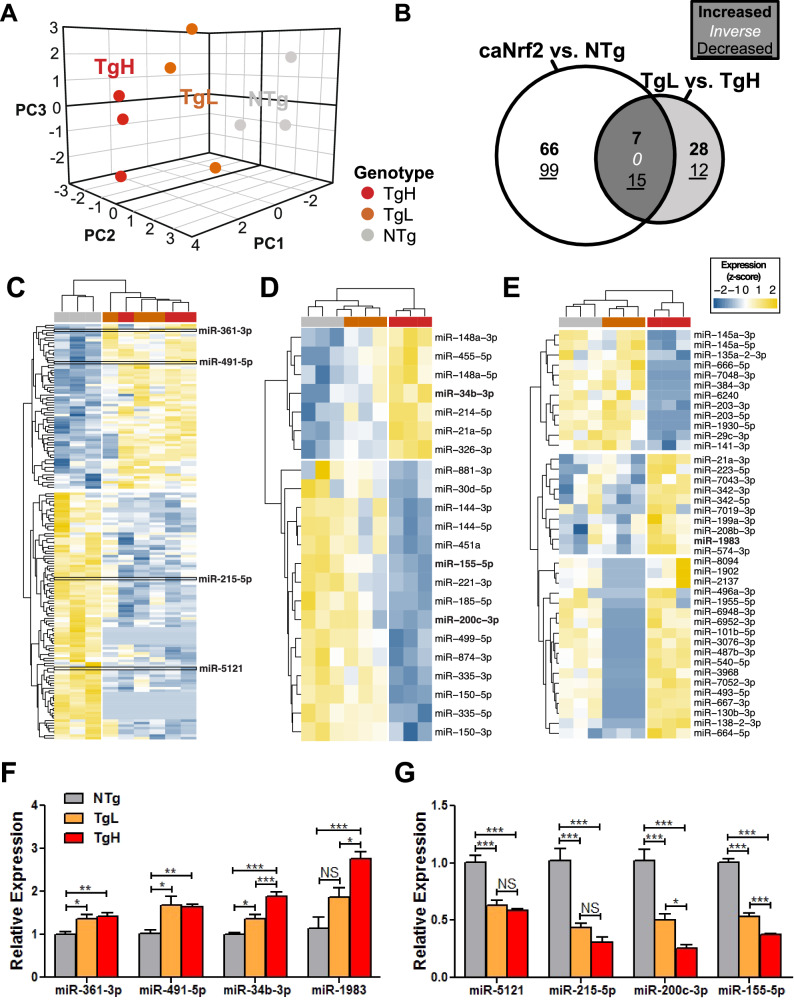
miRNA Sequencing Analysis of ca*Nrf2* Transgene and Dose Effect. (**A**) Unsupervised three-dimensional principal components analysis of rlog-normalized counts from miRNA-sequencing. (**B**) Venn Diagram illustrating the overlap of differentially-expressed miRNAs (DEmiRs) following pooled transgene-and dose–effect analyses (P < 0.05) (n = 3/group). Hierarchial clustering of variance-stabilized counts according to regions illustrated by the venn diagram, with (**C**) 165 “Transgene-only” DEmiRs (Tables S3 and S4), (**D**) 22 “Transgene-Dose” overlapping DEmiRs (Tables S5 and S6), and (**E**) 40 “Dose-only” DEmiRs (Tables S7 and S8). Highlighted in bold are qPCR-validated DEmiRs. (**F**) Real-time quantitative PCR validation of up-regulated and (**F**) down-regulated DEmiRs from among “transgene-only” (↑miR-361-3p, ↑miR-491-5p, ↓miR-5121 and ↓miR-215-5p), “transgene-dose” (↑miR-34b-3p, ↓miR-200c-3p and ↓miR-155-5p) and “dose-only” (↑miR-1983) DEmiRs. Benjamini-hochberg (BH)-adjusted t-test was performed with ****P < 0.0001, ***P < 0.001, **P < 0.01, *P < 0.05 (n = 6–8/group) reported as mean ± SEM.

To determine whether transactivated *Nrf2* expression is associated with differences in global cardiac microRNA expression, we used an unsupervised approach to cluster samples via principal components analysis (PCA) (Fig. 2A). Three distinct clusters emerged from the first 3 eigenvectors (71.7% of cumulative variance), which corresponded exactly to samples from Ntg, TgL, and TgH mice.

To identify dose-responsive miRNA targets of ca*Nrf2*, we performed both a pooled analysis of ca*Nrf2*-Tg vs. NTg (Supplemental Table S1) and a dose-selective analysis of TgH vs. TgL differential miRNA expression (Supplemental Table S2). Comparing the pooled ca*Nrf2*-Tg vs. NTg with TgH vs. TgL analyses via Venn diagram (Fig. 2B, Supplemental Tables S3–S8) revealed three distinct differentially-expressed miRNA (DEmiR) subsets which were designated as follows: (1) “transgene-only” effect (*P* < 0.05 ca*Nrf2*-Tg vs. NTg; left), (2) “transgene-dose” effect (*P* < 0.05 ca*Nrf2*-Tg vs. NTg and TgL vs. TgL; middle), and (3) “dose-only” effect (*P* < 0.05 TgH vs. TgL; right). As anticipated, “transgene-only” DEmiRs displayed equivalent changes across TgL and TgH (Fig. 2C), “dose-only” DEmiRs displayed markedly distinct patterns of expression between TgH and TgL (Fig. 2D,E). The “transgene-dose” DEmiRs reported as significant in each independent analysis are expected to represent direct and dose-dependent targets of ca*Nrf2*, and thus putative effectors of myocardial reductive stress. Several miRNAs within each DEmiR subset were validated via qPCR, supporting their dysregulation in transgenic mouse hearts (Fig. 2F,G).

### *Nrf2*-dependent cardiac mRNA expression

**Figure 3:**
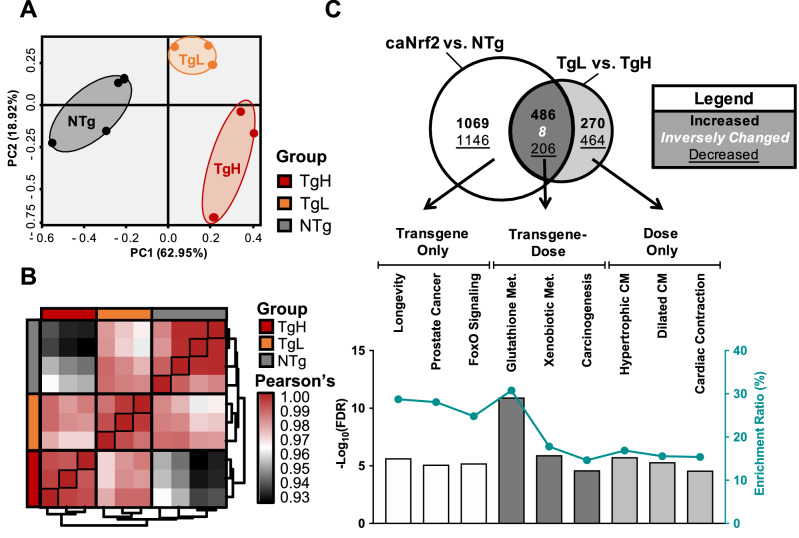
Identification of Direct and Indirect mRNA Targets. (**A**) Unsupervised two-dimensional principal component analysis in R (3.4.2) using normalized counts from non-transgenic (NTg, n = 4), ca*Nrf2* transgenic low (TgL, n = 3) and high (TgH, n = 3) mice. (**B**) Unsupervised correlation heatmap according to pearson’s ρ. (**C**) Venn Diagram illustrating the overlap of differentially-expressed genes (DEGs) following pooled “transgene-only”, “transgene-dose” “dose-only” mRNAs (*P* < 0.05). The underlying bar graph displays FDR-corrected p-values (left y-axis) and gene enrichment ratio (right y-axis) of the top 3 most-enriched KEGG pathways (ranked by p-value) across the venn diagram regions. Enrichment ratio reflects the percentage of a pathway’s genes significantly altered in the current dataset.

In a recent study, we analyzed mRNA-sequencing data from ca*Nrf2* Tg mice to identify differences associated with the physiologic state of myocardial reductive stress^36^. However, this report lacked a comparative analysis of pooled vs. dose-responsive DEGs and the mRNA co-regulatory relationships with miRNAs. Thus, we analyzed differential expression for ca*Nrf2*-Tg vs. NTg (Supplemental Table S9) and TgL vs. TgH (Supplemental Table S10) to relate mRNA dysregulation with miRNA expression patterns. Principal Component Analysis (PCA) (Fig. 3A) and correlation heatmap (Fig. 3B) of DEGs revealed a striking pattern of mRNA expression of TgH compared to TgL mice. Consistent with our functional characterization (Fig. 1) and those of our earlier work^37^, GSEA of “dose-only” mRNA revealed pathways enriched for cardiac pathophysiology while “transgene-only” DEGs implicated noncanonical -possibly indirect- effects of transgenic *Nrf2* expression including “longevity” and “FoxO signaling” (Fig. 3C). Notably, upregulated “transgene-dose” effect DEGs reflected quintessential functions of *Nrf2* signaling such as “Glutathione” and “Xenobiotic Metabolism,” thereby suggesting that this subset of genes represent putative downstream targets of ca*Nrf2* (Fig. 3C). Owing to its role as a positive transcriptional regulator, we were surprised to find that Ca*Nrf2* decreased the expression of 206 “transgene-dose” DEGs (Fig. 3C). Because miRNAs silence gene expression at the post-transcriptional level, we hypothesized that this distinct DEG subset could represent miRNA targets induced downstream of Nrf2 transactivation. These DEGs thus became the focus of our study as putative targets of negative regulation via *Nrf2*-responsive miRNA, or cardiac “reductomiRs”.

### In-silico antioxidant response element (ARE)-based microRNA target prediction

**Figure 4:**
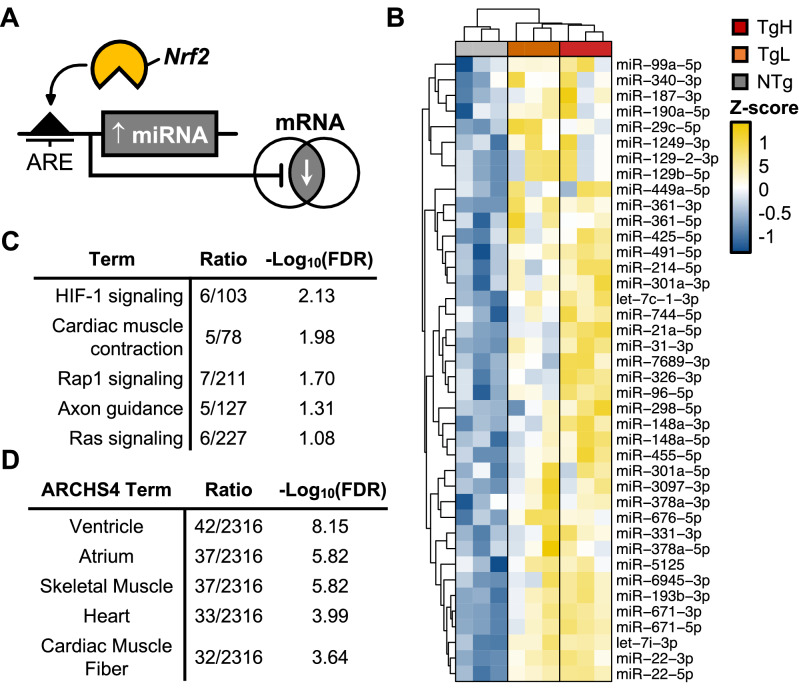
Elucidation of Antioxidant Response Element (ARE)-containing DEmiRs and their Putative mRNA Targets. (**A**) Graphical model representing *Nrf2*-dependent regulation of miRNA and downstream mRNA. (**B**) The 40 induced DEmiRs* (Tg Vs. NTg) containing at least one proximal (+ 5kB) upstream ARE. (**C**) Gene set enrichment (GSEA) of KEGG pathways and (**D**) ARCHS4 gene atlas-based enrichment using DEGs identified as putative targets of ARE-containing DEmiRs. *P < 0.05.

As a transcriptional regulator, *Nrf2* promotes the expression of genes bearing proximal AREs^18^. While GSEA analysis of transgene-dose mRNAs implicated upregulated DEGs as direct targets of *Nrf2*, we hypothesized that the suppression of 206 DEGs in this subset correlates with the relative induction of intermediary miRNAs, as illustrated in Fig. 4A. To identify DEmiRs containing upstream AREs (“ReductomiRs”), we used the position-weighted matrix (PWM) developed by Malhotra et al.^38^ within PWMscan^39^ to identify genome-wide (*Mus musculus*, mm10) *Nrf2* consensus elements. The resulting genomic coordinates were then used to locate a total of 142 AREs within the promoter most-proximal to the coding sequence (+ 5 kB) of 40 upregulated *Nrf2*-dependent DEmiRs (Fig. 4B). From this, approximately half (22/40) of the DEmiRs were found to exhibit a transgene dose-responsive pattern of expression in ca*Nrf2* TgL and TgH hearts. To determine whether ARE bearing dose-responsive miRNAs accounted for downregulated mRNAs within the transgene-dose mRNA subset, we used multiMiR^40^ as a computational tool to systematically compile predicted mRNA targets from the following 8 algorithms: DIANA-microT, ElMMo, MicroCosm, miRanda, miRDB, PicTar, PITA, and TargetScan. Targets were then compiled using cross-algorithm validation to predict miRNA–mRNA interactions^41^. From this analysis, 19 miRNAs exhibited sequence complementarity to seed sequences within 61 down-regulated DEGs. Gene-set enrichment of these DEGs using KEGG pathways disproportionately represented “hypoxia-inducible factor-1α signaling” and “cardiac muscle contraction” (Fig. 4C). Enrichment analysis using the ARCHS4^42^ gene expression atlas revealed a cardiac-specific signature among these DEGs (Fig. 4D). Taken together, these preliminary findings implicate “reductomiRs” as possible *Nrf2*-responsive mediators of myocardial reductive stress.

### Integrative Nrf2-responsive miRNA–mRNA functional network

**Figure 5:**
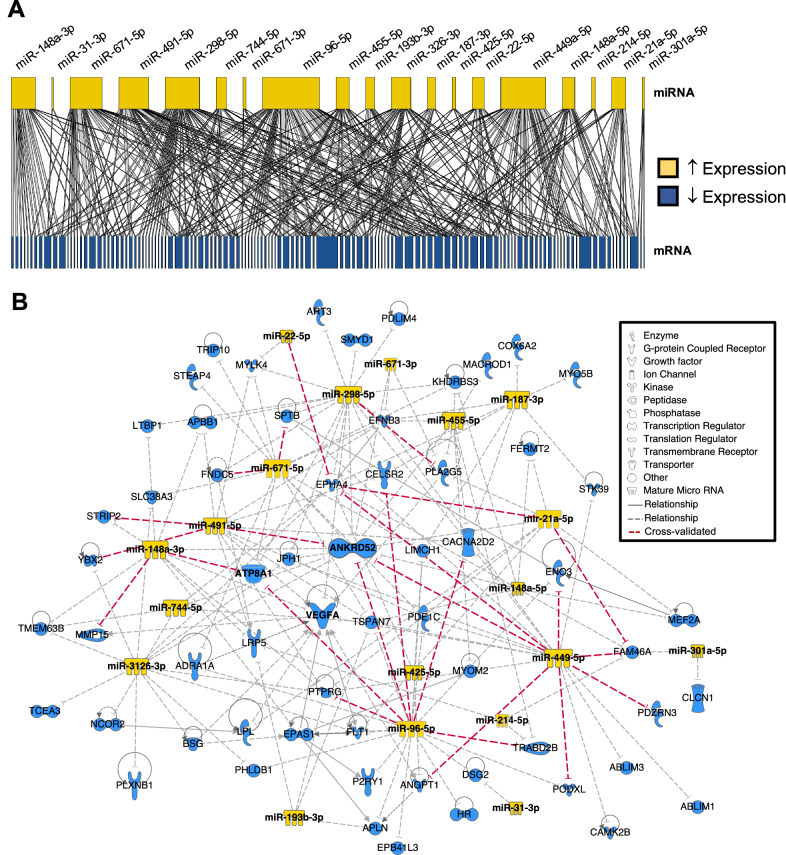
Cardiac miRNA:mRNA Reductive Stress Regulatory Network. (**A**) Bipartite network generated in R (3.4.2) illustrating the complex predicted interaction between ARE-containing induced reductomiRs and suppressed “transgene-dose” effect DEGs, with box-width defined by the relative number of miRNA-mRNA interactions. Line thickness indicates frequency of detection across miRNA target prediction algorithms. (**B**) Ingenuity Pathway Analysis (IPA)-derived signaling network diagram of repressed “transgene-dose” effect DEGs identified as putative downstream targets of ARE-containing DEmiRs. Cross-validated predictions (red dotted lines) using multiMiR (1.12.0) represent miRNA-mRNA interactions predicted by at least 3 independent algorithms.

It became apparent from our in silico analysis that multiple miRNAs shared one or more putative downstream mRNA targets. To understand these interactions further, we generated a bipartite network of the 19 “dose-responsive” reductomiRs (P < 0.01) found to possess a downstream seed sequence among the suppressed mRNAs (Fig. 5A). The resulting network of interactions suggested that *Nrf2*-mediated miRNAs display a varied capacity to influence mRNA transcript abundance in ca*Nrf2*-Tg hearts. Whereas miR-301a-5p and miR-31-3p were predicted to target relatively few mRNAs, the seed sequences of miR-449a-5p, miR-96-5p, miR-298-5p, miR-491-5p, and miR-671-5p matched a larger number of cardiac-specific mRNAs within this network.

Once putative miRNA–mRNA interactions were developed, the curated database from Ingenuity Pathway Analysis (IPA) was used to incorporate known miRNA–mRNA interactions into a ca*Nrf2*-dependent reductomiR–mRNA network, which we then compiled to identify nodal DEGs (Fig. 5B). This approach identified several key mRNAs, which together comprise established mediators of pathological cardiac remodeling: sarcomeric genes myomesin (*Myom2*), myosin light chain kinase 4 (*Mylk4*), voltage gated calcium and chloride channels subunits (*Cacnad2d2, Clcn1*), the striated muscle isoform subunit VIa of cytochrome C oxidase (*Cox6a2*), and adrenergic alpha1a receptor (*Adra1a*). Furthermore, reductomiR targets of ca*Nrf2* were predicted to mediate the negative regulation of SET and MYND domain containing 1 (*Smyd1*) and myocyte enhancer factor 2A (*Mef2a*), two transcriptional regulators essential for cardiac development (Fig. 5B)^43–45^. Although future work should verify direct post-transcriptional regulatory interaction and resultant changes in proteins encoded by these mRNAs, these data implicate miRNAs as putative mediators of *Nrf2* signaling and, ultimately, myocardial reductive stress effectors.

### Bidirectional association of cardiac *Nrf2* and miR-671

**Figure 6:**
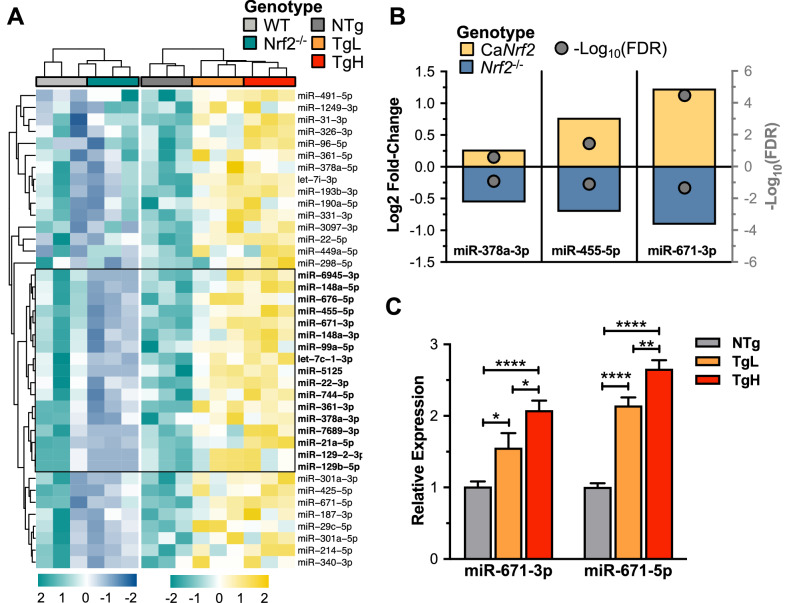
*Nrf2* is both necessary and sufficient to induce reductomiR expression. (**A**) Quantitative realtime PCR (qPCR)-based candidate validation of 3 miRNAs found to reach significance (n = 3, *P* < 0.05) with inverse expression between *Nrf2* knockout (*Nrf2*^−/−^, Table S11) and Ca*Nrf2*-Tg (Table S1) mouse models: miR-378a-3p, miR-455-5p, and miR-671-3p. (**B**) qPCR validation of ca*Nrf2*-Tg miRNA sequencing analysis. Benjamini-hochberg (BH)-adjusted t-test was performed with ****P < 0.0001, ***P < 0.001, **P < 0.01, *P < 0.05 reported as mean ± SEM.

Our data suggest that constitutive activation of cardiac *Nrf2* dose-dependently promotes expression of several ARE-bearing DEmiRs capable of silencing cardiac-specific transcripts. While this result highlights the sufficiency of *Nrf2* for cardiac miRNA induction, it does not indicate which loci require *Nrf2*-mediated transactivation. Therefore, we analyzed the expression of the 40 reductomiRs in hearts from age-matched *Nrf2*^−/−^ mice (Table S11) (Fig. 6A)^22^. Although 17 miRNAs were inversely trending, only three were inversely co-regulated: miR-378a-3p, miR-455-5p, and miR-671-3p (Fig. 6B). Of these, miR-671-3p was the most robustly altered in both animal models. While only miR-671-3p was reported as significant in *Nrf2*^−/−^ miRNA sequencing^22^, both the 5′ (miR-671-5p) and 3′ (miR-671-3p) mature miRNA products of the precursor miR-671 hairpin (pre-miR-671) were significantly increased in ca*Nrf2*-Tg analysis (Fig. 6C). Taken together, these results support that *Nrf2* expression is both necessary and sufficient for the cardiac expression of miR-671.

## Discussion

Since their initial discovery in 1993^46^, two and a half decades of research have shown miRNAs to be central determinants of human biology and disease. As such, miRNA therapies are continuously being patented, and a few have entered clinical trials^47^. The heart’s susceptibility to miRNA dysregulation is well-known, with aberrant miRNA induction found to directly cause pathological cardiac remodeling^48,49^. Nevertheless, endogenous miRNA activity is essential for cardiac morphogenesis^12,13^ and postnatal functions^50,51^. While efforts to identify candidate miRNAs in human patients^14–16^ have led to the identification of key miRNAs governing calcium handling, extracellular matrix remodeling, and vascularization^52^, their role in the regulation of myocardial reductive potential has remained elusive. In this context, our recent discovery of suppressed miRNAs in *Nrf2* knockout (*Nrf2*^−/−^) hearts supports the notion that *Nrf2*-dependent miRNA expression offers a mechanistic link between redox disequilibrium and cardiac pathophysiology^22^.

In the absence of oxidative conditions, *Nrf2* activation is generally inhibited through its interaction with the cytosolic repressor protein Keap1^53–55^. Thus, despite its cardioprotective roles in response to pathological oxidative stress stimuli, such as pressure overload^56^ and ischemia–reperfusion injury^19^, the adaptive phase of *Nrf2* signaling is generally transient in an unstressed adult myocardium. Our laboratory has shown that this basal restriction of *Nrf2* is required, since its sustained activation leads to myocardial reductive stress characterized by accumulation of reducing equivalents and attenuation of physiological oxidant signaling^8,20,21,37^. In addition to our observations in mouse models, others have reported that human germline gain-of-function mutations in *Nrf2* produce early cardiac pathologies^57^. Sariam and colleagues have reported that a subset of human heart failure patients exhibit a hyper-reductive redox state^9^. This suggests that divergent patient populations may contribute to the insufficiency and/or adverse effects of exogenous antioxidant supplementation in cardiac patients^4,5^.

In the present investigation, we observed that constitutive *Nrf2* activation led to profound and dose-responsive miRNA changes in the mouse heart. We specifically found multiple patterns of *Nrf2*-dependent miRNA expression, with 165 “transgene-only” miRNAs, 22 “transgene-dose” miRNAs, and 40 “dose-only” miRNAs were differentially expressed in ca*Nrf2*-Tg mice. Our in silico search for ARE-containing ca*Nrf2*-dependent miRNAs, or “reductomiRs”, identified 40 candidate miRNAs likely to be trans-activated by cardiac *Nrf2*, 22 of which also exhibited dose-responsive induction in TgH and TgL. Lastly, our mRNA target prediction identified 61 putative downstream transcriptional targets for 19 (86%) reductomiRs. While a causal role of these *Nrf2*-mediated reductomiRs remains untested, several miRNAs have already been implicated in the failing human myocardium (e.g. miR-326-3p and miR-214-5p) and plasma (e.g. miR-671-5p)^58,59^. Therefore, this “reductomiR” signature may provide important future insights into redox disequilibrium during cardiac pathophysiology.

Owing to the bioinformatic tools used in this study, mechanistic studies are still needed to confirm our analyses. Since RNA was isolated from heart homogenates, we cannot exclude the possibility that miRNA could originate from non-myocyte cells. Furthermore, future work should differentiate between indirect and effector reductomiRs in myocardial reductive stress as the current investigation does not provide causal evidence that ARE-harboring miRNAs contribute to cardiac pathology or altered redox state.

Nevertheless, our data strongly implicate *Nrf2* as regulator of the myocardial transcriptome, from which direct and indirect regulation of numerous mRNAs and miRNAs are likely to occur. While *Nrf2*-independent changes in the ca*Nrf2*-Tg model remain unexplained, we demonstrate a strong link between *Nrf2*-mediated miRNA activity and post-transcriptional regulation of genes involved in pathological cardiac remodeling. The current study therefore identifies a novel network of Nrf2-responsive miRNAs that amy represent mediators of myocardial reductive stress.

## Supplementary Information

Supplementary Tables.

## Data Availability

All data generated for this study are provided as open-access on NCBI Gene Expression Omnibus (GEO) (miRNA: GSE120087; mRNA: GSE120088), with all coding scripts and quality control available from the following GitHub repository: https://github.com/mepepin/ReductomiRs.

## Abbreviations

*Nrf2*: Nuclear factor, erythroid 2 like 2
Ca*Nrf2*-Tg: Constitutively active *Nrf2* transgenic
TgL: Low-expressing ca*Nrf2* mouse line
TgH: High-expressing ca*Nrf2* mouse line
ARE: Antioxidant response element
DEG: Differentially-expressed genes
DEmiR: Differentially-expressed microRNA
GSEA: Gene set enrichment analysis
